# GIAnT: a Glutamate Imaging Analysis Toolbox

**DOI:** 10.64898/2026.08.07.743580

**Published:** 2026-08-13

**Authors:** Michael E. Xie, Johannes Friedrich, Elizabeth Wirsching, Caleb Jones Shibu, Maedeh Seyedolmohadesin, Naveen Ouellette, Tim Wang, Karel Svoboda, Adam S. Charles, Kaspar Podgorski

## Abstract

Recent advances in fluorescent indicators and optical microscopy now enable *in vivo* synaptic imaging of glutamate, which transmits the majority of signals between neurons in the brain. Extracting fluorescence signals from these recordings is complicated by the minuscule scale and dense clustering of synapses on dendrites, as well as brain motion in behaving animals. Here we present the Glutamate Imaging Analysis Toolbox (GIAnT), a set of automated tools for glutamate imaging data that corrects sample motion, identifies active synapses with super-resolution precision, and extracts synaptic fluorescence signals. Compared to methods designed for cellular imaging, GIAnT reduces motion artifacts, more accurately identifies active synapses, and improves extracted signal quality by reducing contamination from overlapping synapses. By pairing *in vivo* glutamate imaging with *post hoc* expansion microscopy, we find that >70% of the putative synapses extracted using GIAnT matched one-to-one with glutamatergic synapses onto the postsynaptic cell. Our results establish GIAnT as an automated and validated pipeline for processing synaptic glutamate imaging data at scale.

## Full Text Availability

The license terms selected by the author(s) for this preprint version do not permit archiving in PMC. The full text is available from the preprint server.

